# Leveraging comparative genomics to uncover alien genes in bacterial genomes

**DOI:** 10.1099/mgen.0.000939

**Published:** 2023-01-27

**Authors:** Soham Sengupta, Rajeev K. Azad

**Affiliations:** ^1^​ Department of Biological Sciences and BioDiscovery Institute, University of North Texas, Denton, Texas, 76203, USA; ^2^​ Department of Mathematics, University of North Texas, Denton, Texas, 76203, USA

**Keywords:** comparative genomics, phyletic pattern analysis, horizontal gene transfer

## Abstract

A significant challenge in bacterial genomics is to catalogue genes acquired through the evolutionary process of horizontal gene transfer (HGT). Both comparative genomics and sequence composition-based methods have often been invoked to quantify horizontally acquired genes in bacterial genomes. Comparative genomics methods rely on completely sequenced genomes and therefore the confidence in their predictions increases as the databases become more enriched in completely sequenced genomes. Recent developments including in microbial genome sequencing call for reassessment of alien genes based on information-rich resources currently available. We revisited the comparative genomics approach and developed a new algorithm for alien gene detection. Our algorithm compared favourably with the existing comparative genomics-based methods and is capable of detecting both recent and ancient transfers. It can be used as a standalone tool or in concert with other complementary algorithms for comprehensively cataloguing alien genes in bacterial genomes.

## Data Summary

The source code has been made available at https://github.com/sohamsg90/APP-Alieness-by-Phyletic-Pattern. A docker image has been made available at https://hub.docker.com/r/sohamsg90/image_app_v1. All other data are included with the manuscript and supplementary materials.

**Impact Statement**

Bacterial dynamism is shaped by the ability of bacteria to gain new functions by acquiring genes from different lineages through the process of horizontal gene transfer (HGT). It is therefore important to quantify HGT to understand bacterial evolution that impacts different life forms on Earth. Here, we present a new protocol based on comparative genomics to discover foreign genes in bacterial genomes acquired through HGT. The taxonomy-aware framework presented here has the ability to infer both recent and ancient gene transfers. The application of the protocol presented here will spur further research in bacterial genomics and evolution.

## Introduction

Quantification of horizontal gene transfer (HGT) – mobilization of genetic material by means other than vertical (parent to offspring) transmission – is central to understanding prokaryotic evolution [1–4]. Comparative genomics has revealed that these evolutionary events are considerably too frequent in the microbial world to be dismissed as insignificant [5–10]. Several different approaches have been proposed to quantify HGT and understand its extent and impact on prokaryotic evolution [6, 11–15]. Phylogenetic approaches entail reconstruction of gene trees to examine patterns that conflict with the organismal or species tree [11, 16–18] or comparison of genomes of close relatives to examine atypical distribution of genes [19–23]. Composition-based approaches exploit distinct (oligo)nucleotide composition and/or codon usage patterns of horizontally acquired genes, which arise because of their evolution in different (donor) genomic contexts [13, 24–26] . These disparate approaches have complementary strengths and therefore integrative approaches have also been developed to more comprehensively catalogue alien genes in prokaryotic genomes [27, 28].

Because of complementary strengths of different methods [13, 29, 30], it is important to augment the predictive power of each disparate method before combining them within an integrative framework to raise the accuracy bar in alien gene detection. While much efforts have gone into advancing composition-based approaches in recent years, surprisingly, not much attention has been afforded to phylogenetic approaches, particularly those based on phyletic pattern that exploit the distribution of homologues in close relatives to infer HGT, despite the genome databases being more enriched than before; in fact, our survey revealed only three such methods [20–23]. We therefore revisited the phyletic pattern approach with the goal to leverage both the enriched database and the computational efficiency of modern computers to advance the state-of-the-art in alien gene detection via phyletic pattern analysis. In our quest for a robust phylogenetic method for alien gene detection, we developed a new protocol that incorporates taxonomy and a new algorithm that employs blast [31] to efficiently catalogue putative horizontally acquired genes in bacterial genomes. This method, named Alienness by Phyletic Pattern (APP), searches for atypical distribution of a query gene within species, genus and family groups it belongs to. Organisms belonging to these groups with their genomes completely sequenced and available at the NCBI database are enlisted and presented in hierarchical taxonomic groupings for automated phylogenetic analysis by APP. By performing phyletic pattern analysis from lower to higher taxonomic levels (species to family), APP is able to predict both recent and ancient transfer events. An important feature of APP is automated re-analysis of data following an update of the NCBI genome database. Further, owing to taxonomic selection of organisms prior to performing sequence alignment using blast, the computation time is significantly reduced as APP downloads only those genomes that are required for comparison, in contrast to other methods that often require download of an entire nucleotide database prior to analysis. Also, because the procedure is rapid, automated and relatively less resource intensive, it can be performed as often as necessary to update the results.

APP compared favourably with existing comparative genomics-based approaches for alien gene detection, namely IslandPick [23], HGTector [20] and Darkhorse [21, 22], on established benchmarking datasets, as well as on datasets of well-characterized genomes. In what follows, we describe our proposed method, present and discuss our results, and conclude with final remarks.

## Methods

### Genome sequences and annotations

The complete genome sequences and annotations of 18 bacteria, namely *Staphylococcus aureus* subsp *. aureus* MW2 (NC_003923.1) [32, 33], *Staphylococcus aureus* subsp *. aureus* N315 (NC_002745.2) [33, 34], *Staphylococcus aureus* subsp *. aureus* Mu50 (NC_002758.2) [33, 34], *Staphylococcus aureus* subsp *. aureus* COL (NC_002951.2) [33, 34], *Staphylococcus aureus* subsp *. aureus* USA300_FPR3757 (NC_007793.1) [33, 34], *Escherichia coli* CFT073 (NC_004431.1) [35], *Escherichia coli* O157:H7 EDL933 (NC_002655.2) [35], *Salmonella enterica* subsp *. enterica* serovar *Typhi* CT18 (NC_003198.1) [36], *Pseudomonas aeruginosa* LESB58 (NC_011770.1) [37], *Acinetobacter baumannii* AYE (NC_010410.1) [38], *Bordetella petrii* (NC_010170.1) [39, 40], *Burkholderia cenocepacia* J2315 chromosome 1 (NC_011000.1), chromosome 2 (NC_011001.1) and chromosome 3 (NC_011002.1) [41], *Burkholderia pseudomallei* K96243 chromosome 1 (NC_006350.1) and chromosome 2 (NC_006351.1) [42], *Clavibacter michiganensis* subsp *. michiganensis* NCPPB 382 (NC_009480.1) [43], *Mesorhizobium japonicum* MAFF 303099 (NC_002678.2) [44], *Proteus mirabilis* HI4320 (NC_010554.1) [45], *Streptococcus equi* subsp *. equi* 4047 (NC_012471.1) [46], and *Vibrio cholerae* O1 biovar El Tor str. N16961 chromosome I (NC_002505.1) and chromosome II (NC_002506.1) [47], were obtained from the NCBI FTP site (ftp://ftp.ncbi.nlm.nih.gov/genomes-/archive/old_genbank/Bacteria/). These bacteria have been well studied and reported to harbour clusters of alien genes, namely genomic islands (GIs), supported by multiple lines of evidence. The coordinates of GIs and supporting evidence from respective studies are provided in Table S1 (available in the online version of this paper).

Furthermore, comparative assessment of APP was performed on a large benchmark dataset, namely the IslandPick dataset, which was compiled using 118 completely sequenced bacterial genomes by *Langille et al*. [23] (available at https://www.pathogenomics.sfu.ca/islandviewer/download/), and also on a recently curated validation dataset [48] compiled using 104 representative bacterial genomes. Both datasets consist of coordinates of GIs and non-GIs in the respective genomes. The comparative genomics approach used in the construction of these datasets is described in *Langille et al*. [23]. Briefly, using the Mauve 1.2.3 software package [49], pairwise whole genome alignment was performed between a query genome and each of the reference genomes selected based on a pre-computed genome distance matrix using CVTree, in the dataset. For each pairwise alignment, regions >8 kb from the query genome, which remained unaligned, were selected. Furthermore, overlapping regions of the query genome that were not aligned in any of the pairwise genome alignments were subjected to a secondary blast filter (using query genome versus the reference genomes). Any of these regions that had blast hits with coverage of 4 kb or more (i.e. exceeding half of the pre-defined lower bound of island size) were removed and the remaining regions were deemed putative GIs (positive dataset). To construct a non-GI dataset, that is regions not containing any GIs, regions conserved across reference genomes were identified based on the whole genome pairwise alignment by Mauve. These regions were hypothesized to form a stable backbone of the genomes (i.e. lacking horizontally acquired genes) and were used as the non-GI (negative) dataset in the assessment.

### Automated genome selection

**Fig. 1.**
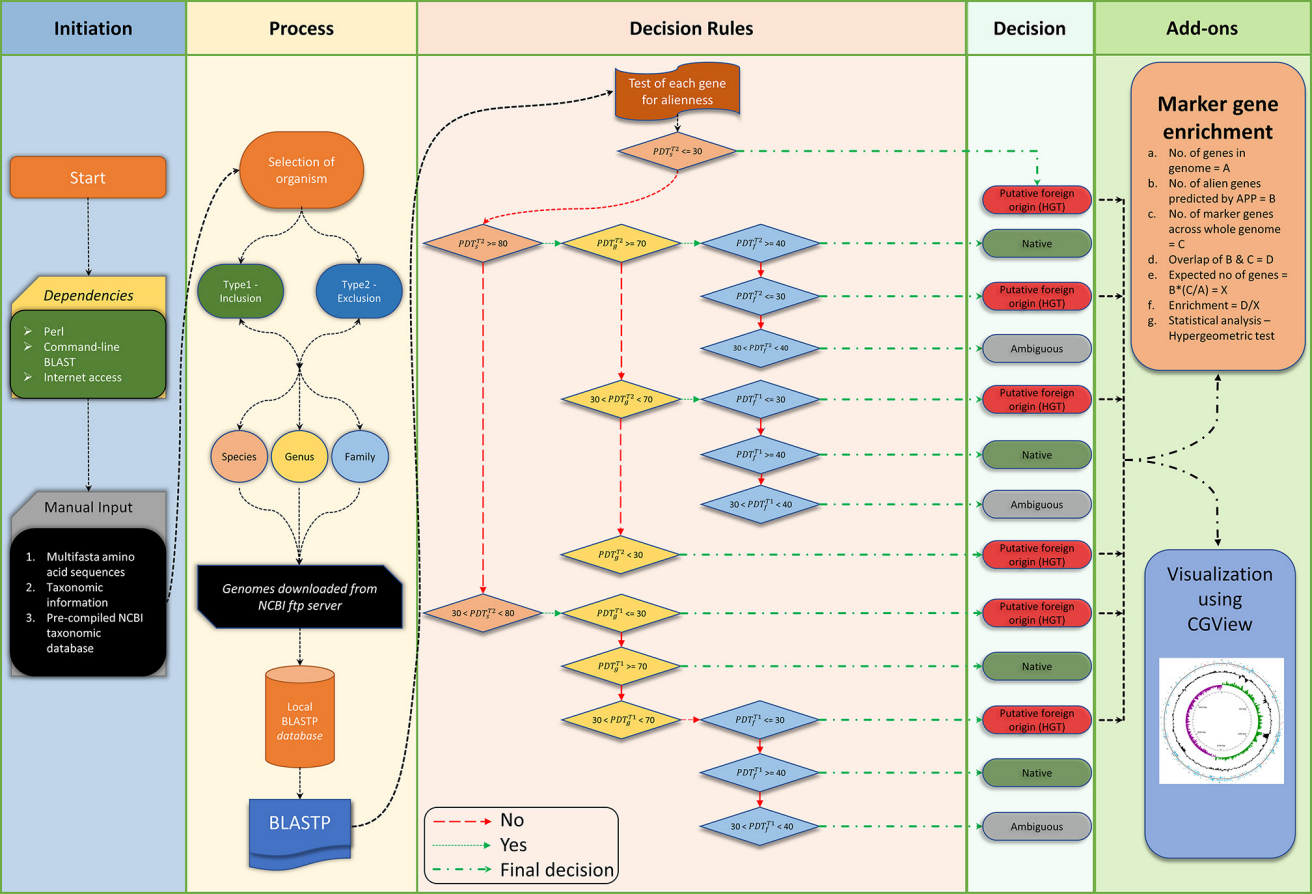
Schematic representation of APP’s protocol for identifying alien genes. The four steps include initiation, process, execution and inference. Initiation entails installing all associated dependencies, followed by constructing a local taxonomic database and inputting query files (multifasta sequence file and taxonomic ID file) to APP. In the second step, APP compiles multiple sequence sets based on the taxonomic rank (species, genus and family) of the query organism for phyletic pattern analysis using blast. For each taxonomic rank of the query organism, two genome datasets were generated: in the first set (Type1; **T1**), all genomes belonging to the taxonomic rank were included, and the second set was obtained by excluding from the first set all genomes that belong to the rank just lower than the rank being assessed (Type2; **T2**). Command-line blastp is performed and the hits with query coverage of 70 % or more and percentage identity above a rank-based cutoff are recorded along with their taxonomic information. The percentage identity cutoff was varied by rank: 60 % for species, 50 % for genus and 25 % for family. For each genome dataset, percentage distribution (PDT) of a query gene was obtained as the percentage of genomes in the dataset that harbour this gene. In the third step, decision rules based on taxonomic rank-specific PDT cutoffs are applied to each of the query genes for annotating it as possibly native or alien (Step 4). Genes that cannot be classified as either native or alien are annotated as ambiguous. Furthermore, the user has the option to perform marker gene enrichment analysis to compile a high-confidence set of putative alien genes among the predicted ones. The alien genes can be visualized in respective circular genome maps using CGView (Step 5: Add-ons). Black dotted arrow denotes the flow of the program. Green arrows arising from diamonds denote ‘yes’, while red arrows denote ‘no’ to test conditions. All diamond-shaped boxes represent different decision rules based on taxonomic rank-specific thresholds. They are colour-coded as per the taxon level under consideration, orange: species; yellow: genus; blue: family. ${PDT}_{s}$ , ${PDT}_{g}$ , and ${PDT}_{f}$ refer to PDT of a query gene in the genome dataset for species, genus and family level respectively. A detailed user manual for installing and running APP has been provided in the GitHub repository at https://github.com/sohamsg90/APP-Alieness-by-Phyletic-Pattern.

The APP architecture (Fig. 1) allows automatic selection of genomes at different taxonomic levels (species, genus and family) for performing sequence comparison using blast (ncbi-blast-2.6.0) to assess the distribution of a gene of interest at each level. APP takes as input a batch file containing fasta sequences along with their NCBI species and taxonomic identities (IDs) (generated using a built-in script or can be obtained from NCBI Taxonomy: https://www.ncbi.nlm.nih.gov/taxonomy). A built-in script is provided to download and set up a local NCBI taxonomic database file consisting of complete taxonomic lineage information of all completely sequenced genomes in the NCBI RefSeq database, along with their FTP links. The taxonomic lineage information (taxon-level names and IDs) was used to compile genome sets at species, genus and family levels. These genome sequences were downloaded to the local machine from the FTP site and a local taxonomy database consisting of sequence information such as genome name, accession number and taxonomic ID was generated for future tracking. Using the above-mentioned sequence sets, the ‘makeblastdb’ command was invoked to generate independent blastp (ncbi-blast-2.6.0) compatible databases.

### Alien gene inference

Alien gene inference was based on the distribution of a query gene in the species, genus and family groups it belongs to. Recent HGT inference was based on the distribution of a query gene in the species group; that is, if the query gene was found absent or sporadically present in the genomes of close relatives within the species group, it was inferred to have been horizontally acquired. If the query gene was found well distributed within the species group but absent or sporadically distributed in the corresponding genus group (excluding the species group it belongs to), it was inferred to have been horizontally acquired in the common ancestor of the species group (i.e. an ancient transfer). Similarly, if the query gene was found well distributed within both the species and the genus groups but absent or sporadically distributed in the corresponding family group (excluding the genus group it belongs to), it was inferred to have been horizontally acquired in the common ancestor of the genus group (i.e. an ancient transfer). If the number of genomes in a taxonomic group was not large enough for a robust inference following the exclusion of genomes of a lower rank group the query gene belongs to, we used all genomes in that taxonomic group for HGT inference. Note that we established multiple thresholds, one for inferring alien genes (present in 30 % or less of close relatives) and taxonomic rank-dependent thresholds for inferring native genes, with rules defined and followed in the order of species to family ranks to identify both recent and ancient HGTs and annotate a portion of the genes that could not be unequivocally classified as alien or native as ‘ambiguous’ genes. The rules and thresholds are specified in the schema diagram shown in Fig. 1. Note that an unusual phyletic pattern may also arise because of gene loss. Here, we have taken a parsimonious approach to address this; that is, if a gene is present in the genome of a bacterium but absent in a large number of its close relatives, gene gain is considered a more plausible explanation than gene loss. We set a high stringency, namely absence from over 70 % of close relatives, to minimize misclassification due to the plausible scenario of gene loss. Being even more stringent could place higher confidence but this may come at the cost of sensitivity, as the acquired genes are likely to become mobilized to few close relatives, and so considering only ‘unique’ gene cases could be underestimating gene gain.

Briefly, if a query gene is present in less than 30 % of genomes of the species group it belongs to, it is classified as alien (recent HGT). On the other hand, if the query was found well distributed in the species group (i.e. in more than 80 % of the genomes), it may still be an alien gene that was acquired in the ancestor of the species group (i.e. an ancient transfer) and therefore we assessed its distribution at higher ranks (we exclude all genomes from a rank just lower than the higher rank being assessed, unless mentioned otherwise) – if the gene is present in less than 30 % of genomes of the genus group it belongs to, it is classified as alien (ancient HGT), and otherwise its distribution is assessed at the family level and is annotated as alien if present in less than 30 % of genomes of the family group it belongs to (ancient transfer). The well-distributed genes within the species group that could not be deemed alien by the tests mentioned above were classified as native if the genes are present in over 40 % of the genomes of the corresponding family group (note that the gene presence criterion was successively relaxed from species to family to account for gene loss and an inability to detect due to divergence over longer evolutionary times), and otherwise the genes were classified as ambiguous. For those genes that are present in the over 30 % but less than 80 % of genomes in a species group, we first considered their presence in the genomes from the corresponding genus group, without exclusion, and classified those present in less than 30 % of the genomes of the genus group as alien while those present in over 70 % of the genomes of the genus group were classified as native; otherwise the assessment was done at the family level, again without exclusion – those present in less than 30 % of the genomes of the corresponding family group were annotated as alien, while those present in over 40 % of the genomes of the family group were annotated as native, and the remaining were then classified as ambiguous. The phyletic distribution thresholds (PDTs, see Fig. 1), that is the cutoffs for percentage genome presence within taxonomic ranks, were inferred based on assessment on the recently published 104-genome benchmark dataset (Bertelli *et al*. dataset) [48], and also by taking cues from the literature on homology inference using blast [36, 50]. APP was calibrated on this dataset, using combinations of thresholds at each taxonomic level (species to family). Performance assessment with different combinations of thresholds is presented in Table S2. The threshold combination yielding the highest overall accuracy was selected to be the default for the program (Fig. 1). Note that by varying these thresholds (except for query coverage kept fixed at 70 %) by 5 % (higher or lower), we found that the performance was not noticeably impacted, thus establishing these thresholds for alien gene detection.

Note that for inferring the presence of a gene in a genome using blastp, a query coverage of 70 % (i.e. at least 70 % of this gene should be aligned against a blast hit) was used across all taxonomic ranks (from species to family); however, the percentage identity cutoff was varied by ranks: 60 % at species, 50 % at genus and 25 % at family levels, in order to account for increasing divergence with higher ranks.

### Marker gene enrichment

Post-hoc analysis entailed assessment of predicted alien gene sets for enrichment of features or markers typically associated with mobile genomic elements. This add-on to APP uses HMMER [51, 52] to annotate markers using a custom Pfam database [53] constructed as described previously [54]. Briefly, the custom database was built by performing a search for GI-specific markers such as transposase, integrase, prophage, recombinase and plasmid. Additionally, the database was manually curated to eliminate any profile HMMs not representing GI markers. This resulted in a local database of ~450 profile HMMs representing marker gene families [54]. Genes from a genome of interest were probed against this database and those with ‘hits’ in the database with expected values 0.01 or less [55] were annotated as marker genes. A hypergeometric test was conducted to assess the statistical significance of enrichment of markers within the predicted alien gene set (Fig. 1).

### Visualization

As an add-on to APP is a local version of the CG Viewer program enabling visualization of alien genes in their respective circular genome maps generated by CGView [56] (Fig. 1). As an example, we provide in Fig. S1 a circular genome map depicting locations of alien genes predicted by APP in the *Staphylococcus aureus* Mu50 genome.

### Comparative assessment with existing phyletic pattern-based methods for alien gene detection

APP was compared with current phyletic pattern-based methods for alien gene detection at their default parameter settings, namely IslandPick [23], HGTector [20] and Darkhorse [21, 22]. IslandPick uses CVTree [57] to select genomes for alignment in order to delineate GIs and native regions. HGTector is a blast-based approach that first performs all-against-all blastp alignments and then a weight distribution review of all hits grouped by phylogenetically informed categories. Darkhorse performs a low-stringency blast search against a reference database, followed by calculation of a lineage probability index (LPI) score and an optional combination with phylogenetic tree-building to detect alien genes.

### Assessment metrics

The standard performance metrics, recall (sensitivity), precision and F-measure, were used for performance assessment. As the evaluation datasets comprised GI-borne genes, we tested the ability of APP and other phyletic pattern-based methods to identify island genes. We therefore defined recall as the percentage of island genes in a test dataset or genome that were correctly identified by a method. Precision is defined as the percentage of predicted alien genes that were correct, that is were island borne genes. F-measure is an accuracy metric that incorporates both recall and precision, and is defined as the harmonic mean of recall and precision:

$$F-measure=\frac{2∙Recall∙Precision}{Recall+Precision}$$

Note that for the performance assessment, all genes deemed ‘ambiguous’ by APP were considered native. In addition to these metrics, we have also provided the confusion matrices that were used in computing these metrics (Table S6).

## Results and discussion

### Method overview

The schematic diagram of the workflow of APP is shown in Fig. 1. APP takes as input two files – a multi-FASTA file of protein sequences and a file containing the taxonomic and species IDs of the organisms. APP outputs a list of putative horizontally acquired genes along with the information of being possibly recent or ancient transfers. Users can also perform marker gene enrichment. Furthermore, as an add-on to APP is a local version of the CG Viewer program enabling visualization of putative alien genes in their respective circular genome maps generated by CGView [56] (Fig. 1).

The process begins with an automated selection of genomes to compile taxon-specific genome sets as described in the Methods. blastp-compatible databases are constructed using the ‘makeblastdb’ command for each of the genome sets. We considered only completely sequenced and assembled genomes for phyletic pattern analysis. In addition to the general workflow of APP (Fig. 1), we provide in Fig. S3A APP’s workflow for an example of a gene deemed native and in Fig. S3B, an example of a gene deemed alien, with cutoffs also indicated.

### Comparative assessment on genomes with known genomic islands

**Fig. 2.**
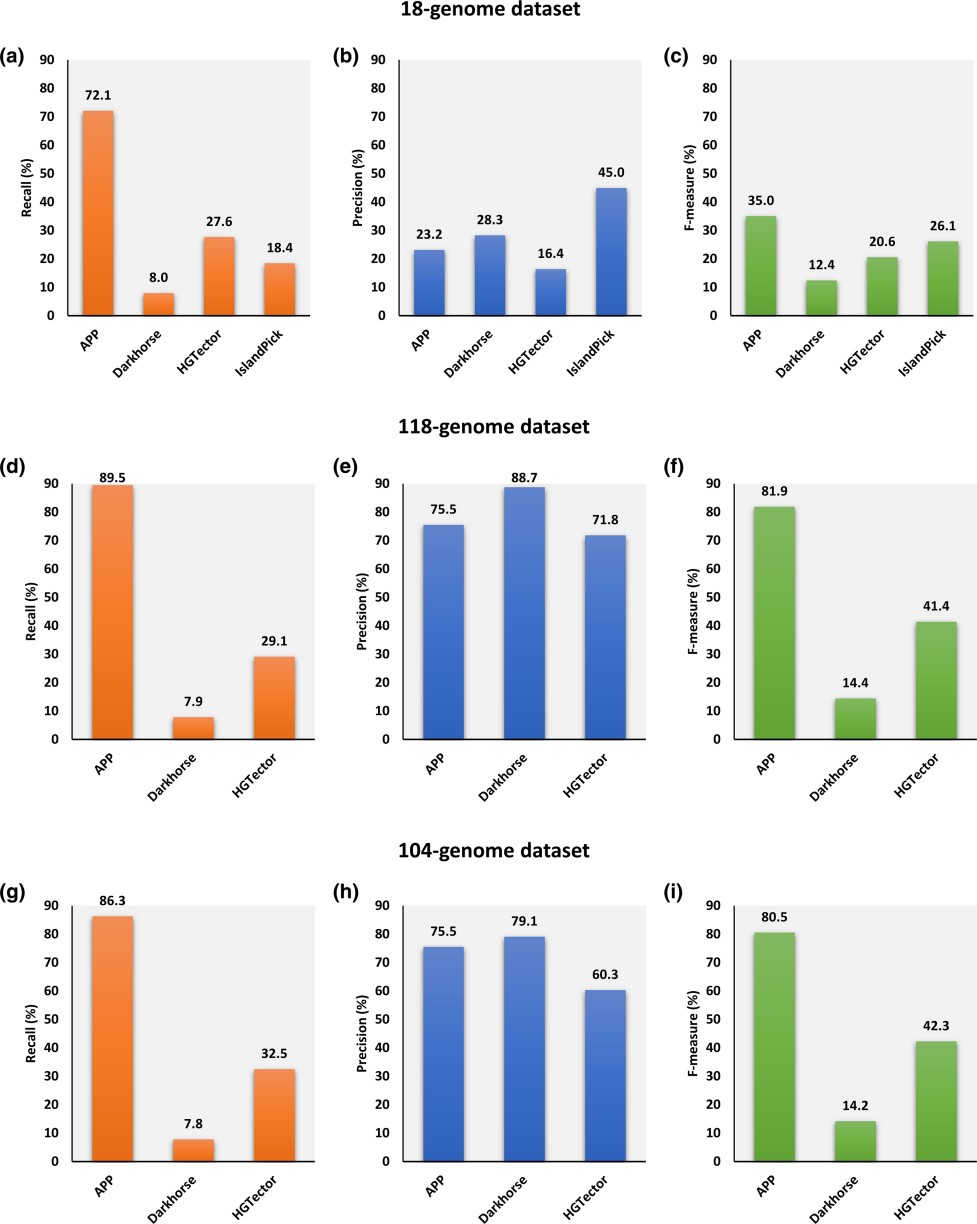
Performance assessment of phyletic pattern-based alien gene detection methods on different datasets. The performance of APP was assessed against current phyletic pattern-based methods, Darkhorse, HGTector and IslandPick, on 18 genomes with known islands (**a–c**), 118-genome dataset (**d–f**), and 104-genome dataset (**g–i**). Performance was assessed in terms of recall, precision and F-measure. For the 118-genome and 104-genome datasets, IslandPick was excluded from the performance assessment as these datasets were compiled using IslandPick. The Methods section gives details on datasets and performance metrics.

The performance of APP was assessed against the current phyletic pattern-based methods, namely HGTector, Darkhorse and IslandPick, on 18 genomes with known islands (see Methods; Fig. 2a–c). These 18 genomes have a total of 7 177 alien genes harboured on 164 GIs (also referred to as 18-genome dataset). APP was able to identify 5 175 of these alien genes, rendering a recall of 72 %, highest among all the methods (Fig. 2a). The next best performing in recall was HGTector with a recall of 27 % (1 987 alien genes correctly identified). These were followed by IslandPick and Darkhorse, producing relatively low recall of values of 18 and 8 % respectively (Fig. 2a). IslandPick had the highest precision of 45 %, followed by Darkhorse and APP with 28 and 23 % respectively (Fig. 2b). HGTector had the lowest precision of 16 % (Fig. 2b). We then assessed performance based on the overall accuracy metric, F-measure, which is the harmonic mean of recall and precision. APP produced the highest F-measure of 35 %, outperforming IslandPick by ~9 %, HGTector by ~14 % and Darkhorse by ~22 % (Fig. 2c). The relatively high precision of IslandPick was expected as it was designed to be highly precise in order to generate high-confidence datasets of alien and native genes for use in benchmarking of GI prediction methods [23]. However, it suffers from low recall, and as a consequence, yields low overall accuracy (F-measure). Darkhorse takes as input ‘self-definition’ terms and uses them to make the blast search more stringent, thus attempting to minimize false positives [21, 22]. This does not, however, render an overall accuracy better than that of APP due to a very low recall of alien genes. HGTector suffers from both low recall and low precision. APP could attain the highest overall accuracy due to relatively high recall.

Although the precision by APP was low, it could be attributed, in part, to incomplete annotation of alien genes in these genomes. Alien genes harboured on GIs are only annotated, while those outside of GIs are not. The latter, as yet unknown alien genes, were labelled as native for the purpose of evaluation. Furthermore, the list of GIs in each genome is probably not exhaustive and therefore a substantial number of alien genes harboured by as yet unknown GIs might also have been mislabelled as native in this assessment procedure. It is plausible that many of the ‘false positives’ by APP are actually true positives. This may be true for other methods as well, but note that methods such as IslandPick are highly conservative and do not tend to generate ‘false positives’, but because of this, they suffer from false negatives as is obvious from their inability to detect many well-supported GIs or alien genes. Although this dataset does not serve as an ideal dataset for benchmarking, it does provide the most comprehensive set of GI-borne alien genes to date, which could be, and has often been, used for assessing the relative performance of different methods [36, 37, 54, 58]. Notably, APP, GI agnostic by design, detected GI-harboured genes better than methods (e.g. IslandPick) that were designed to identify GIs and thus the associated alien genes. We further examined the sets of false positive genes in these genomes for marker gene enrichment. While there was some level of enrichment of marker genes in these sets in all of the 18 genomes, 11 were found to be significantly enriched (*P*<0.05) (Table S7). This supports our hypothesis that many of the false positives could indeed be true positives. We investigated the ambiguous genes: those that could not be classified as native or alien by APP. In total, 1870 (~2 %) of 70 400 genes were annotated as ambiguous across the 18 genomes. Of these, only 144 genes were found to be marker genes (Table S8).

Genome-wide assessment showed that APP outperformed other methods on 11 of 18 genomes (total of 22 chromosomes) in terms of overall accuracy (F-measure). APP’s F-measure of 57 % on *Salmonella enterica* CT18 was the highest attained by any method on these test genomes (Table S3). Darkhorse failed to identify any alien genes in *Salmonella enterica* CT18 and *Streptococcus equi* 4047, and IslandPick could not identify any genes in *Staphylococcus aureus* MW2, *Staphylococcus aureus* COL, *Burkholderia cenocepacia* J2315 chr 3, *Bordetella petrii *, and *V. cholerae* N16961 chr I and II (Table S3). APP and HGTector were the only two methods that predicted alien genes in all the 18 test genomes (Table S3).

**Fig. 3.**
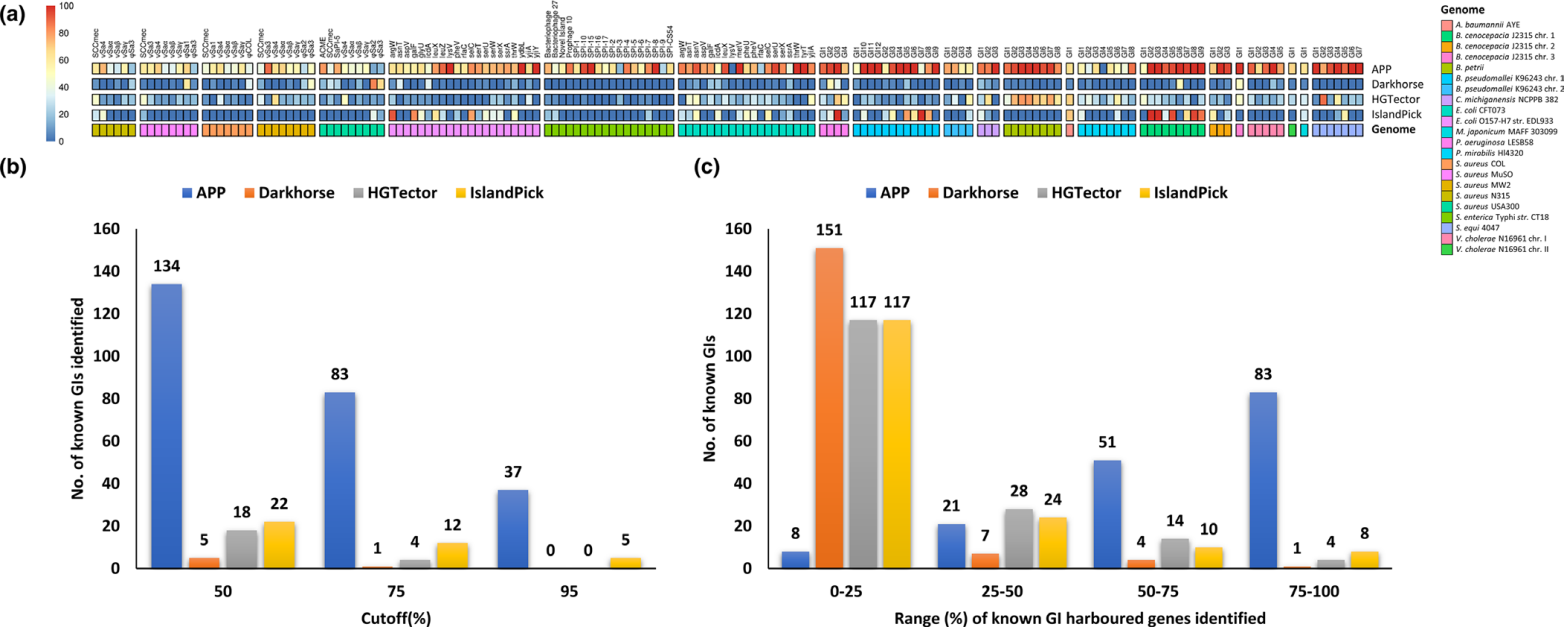
Known genomic islands identified. (**a**) Heatmap showing the percentage of GI-harboured genes identified by different methods, for each GI in 18 well-characterized genomes. Names of GIs in different genomes are shown on the top. On the rightmost side, the name of each method is shown and on the leftmost the heatmap scale is displayed. The heatmap scale represents the percentage of GI-harboured genes correctly identified by each tool. (**b**) Number of known GIs identified at a certain cutoff by a method. A cutoff of *X *% means that *X *% of genes in a GI are identified as alien in order for the GI to be deemed identified. (**c**) Number of known GIs falling within a range (e.g. 0–25 %) of island-harboured genes identified by a method. The names of the strains for which the data are shown in (a)–(c) are shown on the right of (a).

At the default settings of the programs, we assessed their ability to detect known GIs at 50, 75 and 95 % cutoffs, where *X *% cutoff means that *X *% or more of the genes harboured on an island should be identified as alien in order for the prediction to be deemed a success (i.e. the island called correctly by a method). X≥50 implies that a majority of island-harboured genes must be identified for the prediction to be deemed a success. At a 50 % cutoff, APP identified 134 of 164 islands (~81 %), whereas IslandPick, HGTector and Darkhorse identified only 22, 18 and five islands respectively. At more stringent cutoffs (75 and 95 %), fewer islands were detected by APP (83 and 37, respectively), while these numbers were significantly lower for the other methods (Fig. 3b). For each method, we computed the number of known islands falling within each of the ranges 0–25, 25–50, 50–75 and 75–100 % of island-harboured genes identified by the method. In the case of APP, the known GIs fell mainly in the upper ranges, 50–75 and 75–100 % (Fig. 3c), indicating that APP has the ability to identify islands by virtue of classifying a majority of island-harboured genes as alien. On the other hand, among all ranges, HGTector, Darkhorse and IslandPick had the highest count of known GIs in the 0–25 % range, indicating their inability to detect most of the known islands (Fig. 3c). Notably, only two GIs had none of their genes classified as alien by APP, in contrast to 103 for IslandPick. Fig. 3a) summarizes this analysis in the form of a heatmap.

Results from assessment on whole genome datasets highlight the superior performance of APP relative to other comparative genomics methods designed to identify alien genes based on phyletic pattern. However, as these genomes may not have been exhaustively studied for the presence of GIs, they may not serve as ideal benchmark datasets, although they have been used in successive studies for relative comparative assessment due to a paucity of validation datasets. Langille *et al*. [23] and Bertelli *et al*. [48] realized this problem and attempted to address it by compiling large benchmark datasets from 118 genomes and 104 genomes respectively. For comprehensive comparative assessment, we therefore used these datasets for benchmarking of APP and other programs as discussed below.

### Assessment on large benchmarking datasets

Langille *et al*. [23] compiled a 118-genome dataset (often referred to as IslandPick dataset) containing high-confidence GIs and non-islands in each of the 118 genomes considered in their study. Since this dataset was compiled using IslandPick, we did not include IslandPick in the comparative assessment here. Note that a highly conservative approach was taken to compile a set of islands and another set of non-islands; these are, therefore, considered as high-fidelity benchmarking data and have been used for evaluation of island prediction methods in several subsequent studies [23]. We assessed the performance of APP, Darkhorse and HGTector on this dataset containing positive (island) and negative (non-island) examples assembled from 118 prokaryotic genomes (Fig. 2d–f).

APP produced the highest recall of 89 %; in contrast, the recall values of HGTector and Darkhorse were very low at 21 and 7.8 %, respectively (Fig. 2d). All three methods generated precision of over 70 %, with Darkhorse being the most precise at 88 %, followed by APP at 75 % and HGTector at 71 % (Fig. 2e). As APP balanced recall and precision much better than the other methods, its overall accuracy of 81 %, assessed through F-measure, was much better than that of HGTector (41 %) and Darkhorse (14 %) (Fig. 2f).

Bertelli *et al*. [48] recently compiled a curated dataset of high-confidence GIs and non-islands in 104 genomes. Similar to Langille *et al*.'s study [23], this dataset was also compiled using IslandPick, and therefore we did not include it in the comparative performance assessment. Similar to the evaluation on the 118-genome dataset (IslandPick dataset), APP produced highest recall of 86 % and Darkhorse had the highest precision value of 79% (Fig. 2g, h). HGTector and Darkhorse produced low recall values of 32 and 7 %, respectively (Fig. 2g). APP balanced recall and precision well, yielding an F-measure of over 80 %, substantially higher than the 14 and 42 % generated by Darkhorse and HGTector, respectively (Fig. 2i).

Phylum-wise performance assessment was performed using the 104-genome dataset (Bertelli *et al*. dataset [48]). We considered the three major phyla represented in this dataset, namely *Actinobacteria *, *Firmicutes* and *Proteobacteria* (Fig. S2). APP produced the highest recall of 96 % for *Actinobacteria *, 94 % for *Firmicutes* and 84 % for *Proteobacteria* (Fig. S2A). Darkhorse, on the other hand, had the highest precision of 96, 48 and 91 % for these phyla respectively (Fig. S2B). Consistent with the overall performance in identifying alien genes (Fig. 2i), APP balanced recall and precision well for each of these phyla, yielding overall accuracies (F-measure values) of 95, 49 and 85 % for *Actinobacteria *, *Firmicutes* and *Proteobacteria,* respectively, substantially higher than those by Darkhorse and HGTector (higher by 28–74 and 19–48 %, respectively, Fig. S2C).

### Assessment on synthetic genomes

The performance of APP was also assessed on synthetic genomes at different taxonomic levels. Synthetic genomes provide a test dataset where the evolutionary history of each gene is known. We used *Staphylococcus aureus* MW2 as the recipient genome. The genomes of *Alkaliphilus metalliredigens* QYMF (NC_009633.1), *Erysipelothrix rhusiopathiae* str. Fujisawa (NC_015601.1), *Macrococcus caseolyticus* JCSC5402 (NC_011999.1) and *Salinicoccus halodurans* H3B36 (CP011366.1) represented a broad range of phylogenetically distinct donors. *M. caseolyticus* and *Salinicoccus halodurans* represent the genera *Macrococcus* and *Salinicoccus,* respectively, within the family *Staphylococcaceae* that the recipient *Staphylococcus aureus* belongs to. *A. metalliredigens* and *Erysipelothrix rhusiopathiae* represent the classes *Clostridia* and *Erysipelotrichi,* respectively, within the phylum *Firmicutes* that *Staphylococcus aureus* belongs to [58]. First, ten genes, with at least two orthologes in the recipient genome [59], were selected from each of the donor genomes *M. caseolyticus* and *Salinicoccus halodurans* and transferred into the *Staphylococcus aureus* MW2 genome, with increasing genetic divergence (0, 5, 10, 15, 20, 25 and 30 %) simulated using HgtSIM [60]. As a result, HgtSIM produced seven simulated genomes with alien gene acquisitions from *Macrococcus* and *Salinicoccus *. Similarly, seven more synthetic genomes were constructed with acquisitions from *Clostridia* and *Erysipelotrichi *. Due to the limitation on choice of input file format, simulated genomes could only be used with APP and HGTector. APP identified all genes that were transferred into the recipient genome (Table S4), which can be attributed to its taxonomy guide design to assess gene distribution successively from species to family levels. Note that as the already resident alien genes in the *Staphylococcus aureus* MW2 genome could not be unambiguously identified and removed, APP might have correctly classified these as well (some if not all), but these were considered native in the assessment, which could have undermined its overall accuracy (F-measure). HGTector could identify only 38 % of transferred genes from *Macrococcus* and *Salinicoccus* and 7 % from *Clostridia* and *Erysipelotrichi *, rendering an overall accuracy (F-measure) of 55 and 13 %, respectively (Table S4).

### Hierarchical taxonomic rank-guided augmentation in APP’s performance

**Fig. 4.**
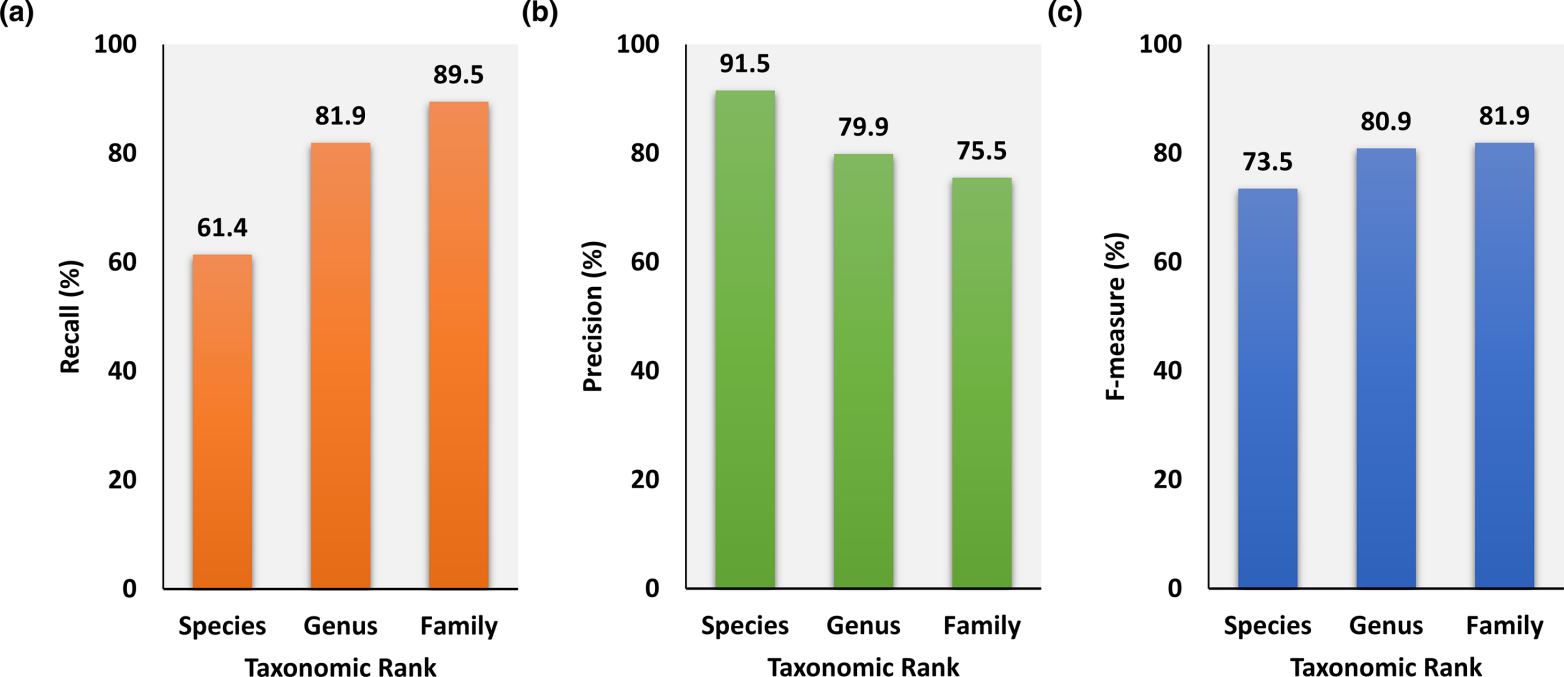
Assessment of APP’s performance at different hierarchical taxonomic levels (species to family). APP begins with the species-level phyletic distribution information and then progresses towards the higher hierarchies in taxonomy, namely the genus and family levels. Higher level analysis was intended to augment APP’s performance by identifying ancient HGTs. Taxonomy-guided augmentation of overall accuracy (F-measure) in identifying alien genes in the 118-genome dataset is shown on the right, with respective Recall shown on the left and Precision in the middle.

The phyletic pattern analysis begins at the species level and then advances to higher hierarchy in taxonomy, namely the genus and family levels. Our hypothesis was that this hierarchical taxonomic rank-guided analysis will augment APP’s performance as the analysis advances from lower (species) to higher ranks (genus and family), by accounting first for recent transfers (species-level analysis) and then for ancient transfers (genus- and family-level analyses). We assessed augmentation in APP’s performance as the phyletic analysis progressed from species to genus, and then to family level. We first performed this assessment on the 118-genome dataset [23] as both positive (alien) and negative (native) sets are considered to be high-fidelity. On this dataset, APP attained a recall of 61 %, precision of 91% and F-measure of 73 % at the species level (Fig. 4a). The sensitivity of 61 % indicates that a majority of the alien genes were identified at the species level; this is not unexpected as a large number of alien genes in a genome at any snapshot of time are probably recently acquisitions, as most acquired genes do not confer long-term benefits and are eventually lost from genomes [61, 62]. The precision of 91 % indicates minimal false positives and so a high-fidelity prediction set (Fig. 4b). We expect the recall to increase with genus-level analysis, as some of genes that were found well distributed in the species genomes could have been acquired horizontally in the common ancestor of the species group. Furthermore, some of the genes that were deemed ambiguous at the species level could be reclassified as alien or native based on additional information retrieved at the genus level. We indeed observed, as expected, an increase in recall by over 20 % at the genus level (Fig. 4a). This did, however, result in a decrease in precision though disproportionately by only ~10 %, and therefore the overall accuracy (F-measure) increased by over 8 % (Fig. 4c). The next higher level (family) analysis rendered a further increase in recall by over 9 %, for the similar reasons as for the increase at the genus level, that is by identifying ancient transfers (those that were acquired in the common ancestor of each of the genus groups) or by resolving the status of as yet ambiguous genes. The decrease in precision was again disproportionately lower, by ~4 %, thus rendering an augmented overall accuracy (an increase of ~1 %). A similar trend was observed when APP was assessed on the updated, 104-genome benchmark dataset (Bertelli *et al*. dataset [48]) (Table S5). Thus, information from each taxonomic level contributed to the overall performance of APP.

### Conclusions

Rapidly growing databases of genomes call for timely update of current approaches for comparative genome analysis or development of new approaches to genome analysis. Here, we revisited comparative genomics for alien gene detection and developed a new taxonomy-guided approach that leverages information-rich genome databases to augment the power of comparative genomics in alien gene discovery. Comparative assessment demonstrated the overall superior performance of our method APP over current phyletic pattern-based methods. APP can be used as a standalone program or in concert with other, complementary approaches, such as those based on phylogenetic tree reconstruction or sequence composition, to comprehensively catalogue alien genes in bacterial genomes.

### Software availability

The source code of APP is scripted in Perl and has been made publicly available at https://github.com/sohamsg90/APP-Alieness-by-Phyletic-Pattern, where details on the usage and computational dependencies are provided. A docker image has been also provided and detailed instructions are made available at https://hub.docker.com/r/sohamsg90/image_app_v1.

## Supplementary Data

Supplementary material 1

Supplementary material 2

Supplementary material 3

Supplementary material 4

Supplementary material 5

Supplementary material 6

Supplementary material 7

Supplementary material 8

Supplementary material 9
