# Supplementary material for: Leveraging comparative genomics to uncover alien genes in bacterial genomes
Source: Microb Genom. 2023 Jan 27;9(1):mgen000939. doi: 10.1099/mgen.0.000939 (PMC9973850; doi:10.1099/mgen.0.000939)
Supplement: Supplementary material 1 [file mgen-9-939-s001.pdf]

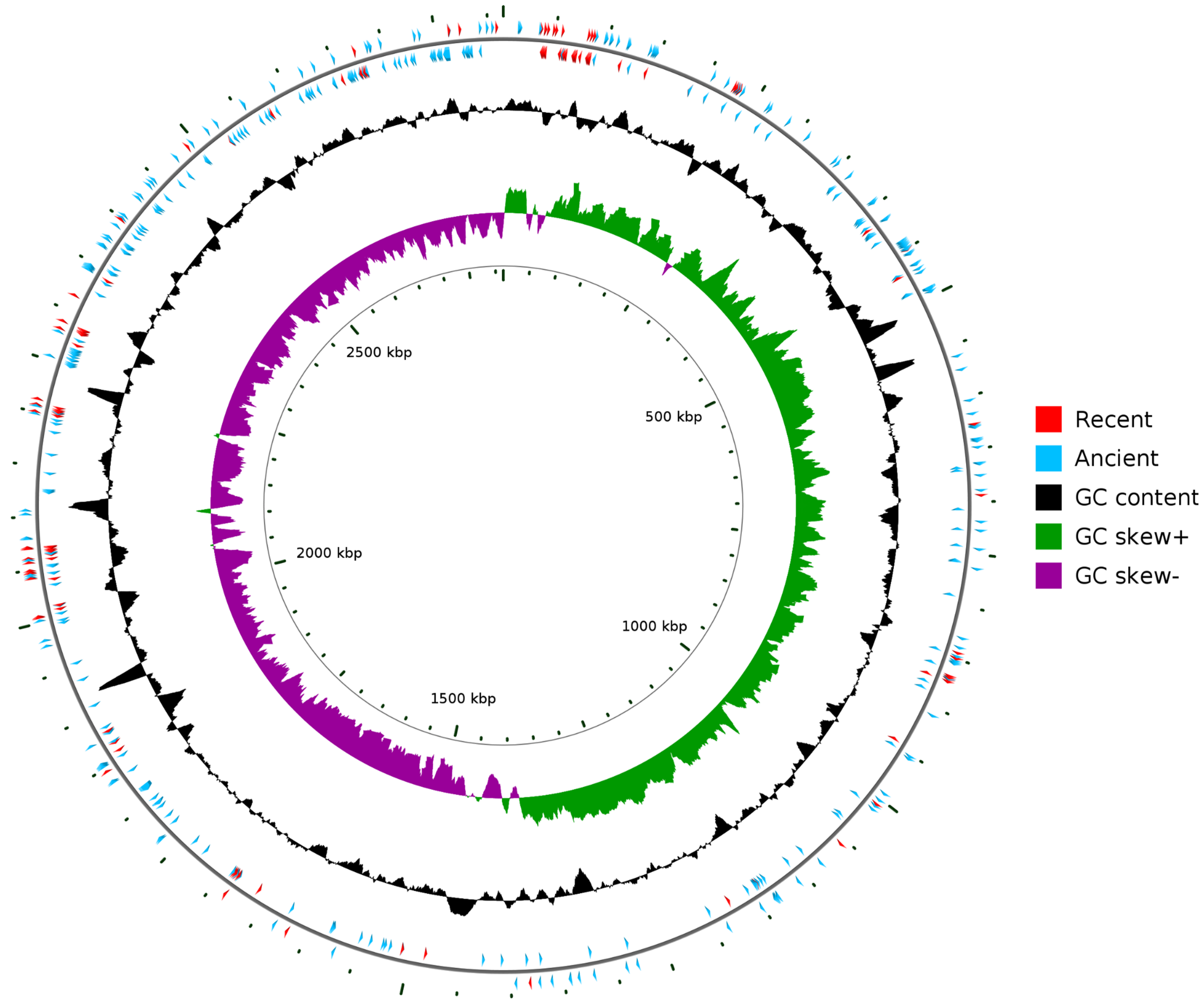


**Supplementary Figure S1: Circular genome map of *S. aureus* Mu50 (NC_002758)**. Alien genes detected by APP are represented in the form of circular genome map with CGView tool provided as an add-on to APP. The outermost circle represents the '+' strand, while the next circle represents the '-' strand. Genes found to be procured by horizontal gene transfer are shown as recent (Red) and ancient (Blue) transfer. The next circle shows the GC-content followed by GC-Skew of the genome. The name of the organism is provided in the center.

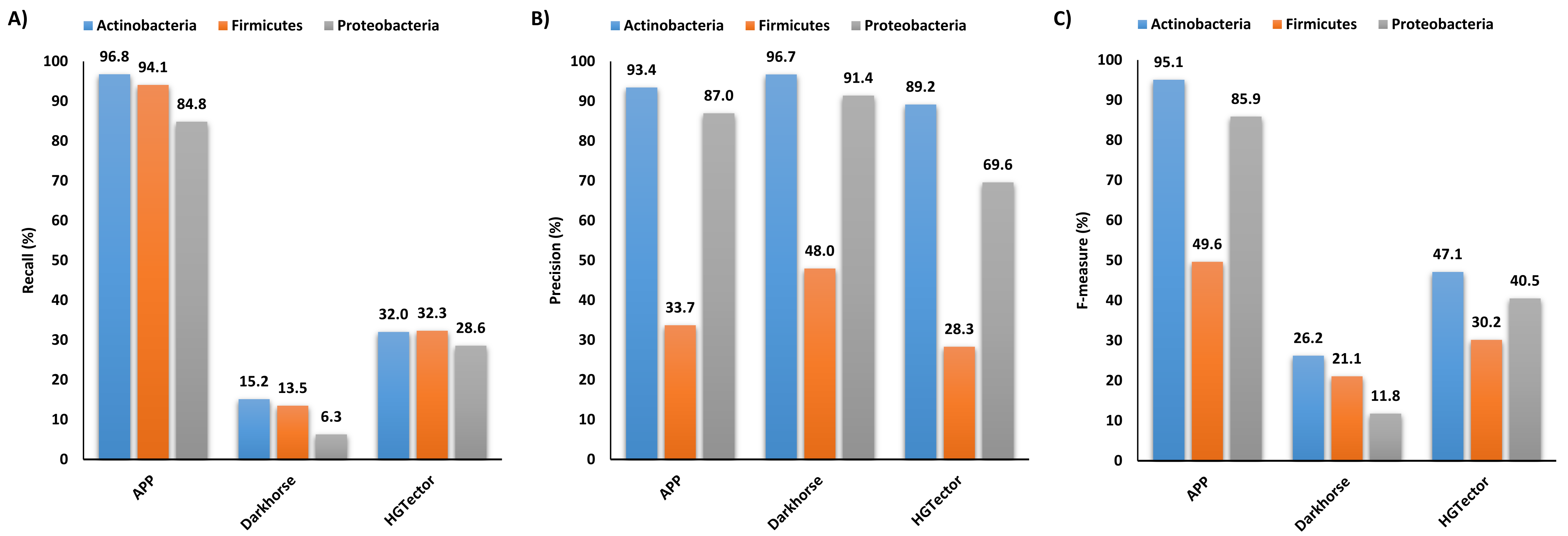


**Supplementary Figure S2:** Performance assessment of alien gene detection methods across different phyla (A-C) on the 104-genome dataset. Performance was assessed in terms of recall, precision, and F-measure.

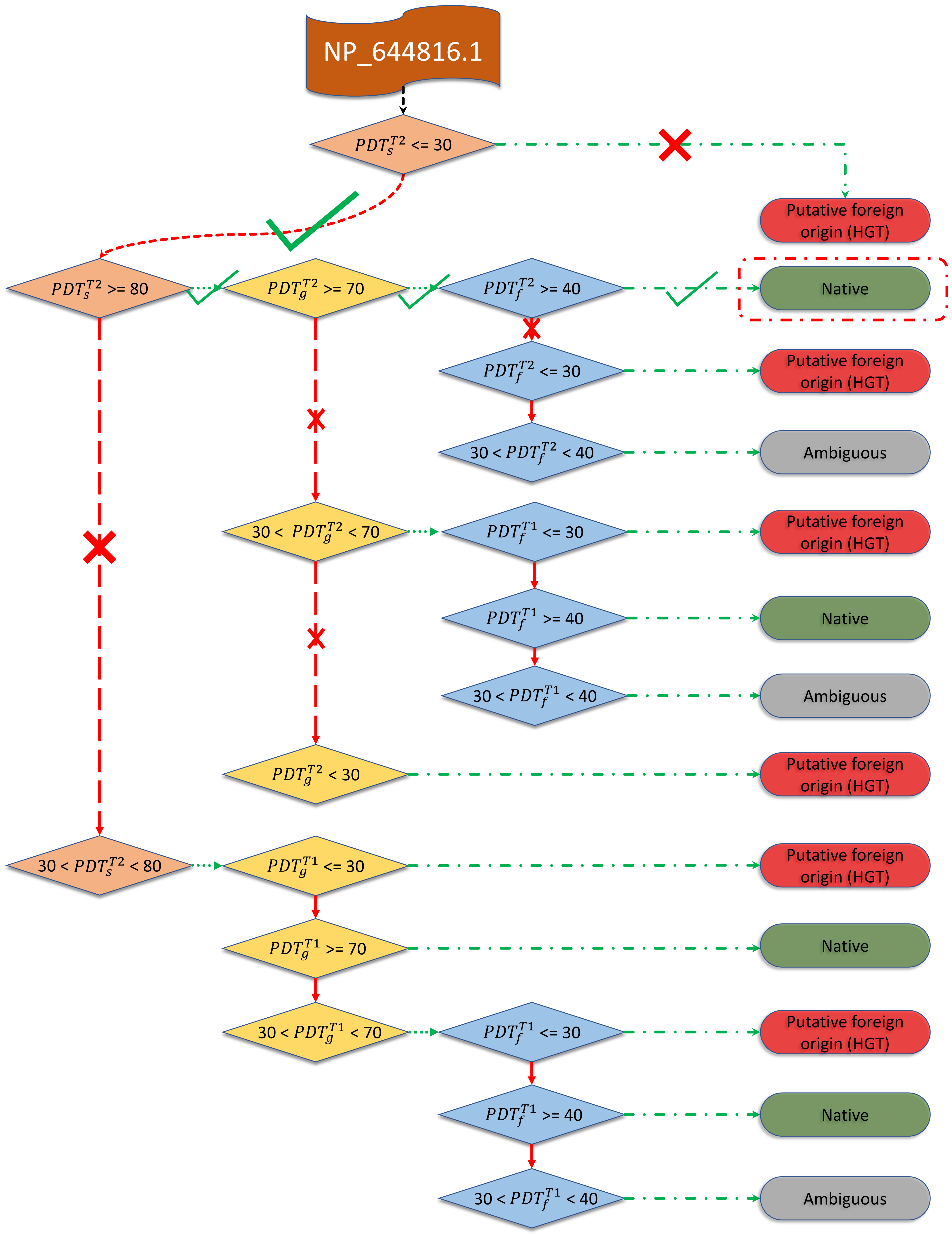


| Phyletic Distribution Threshold (PDT) | NP_644816.1 |
|---|---|
| PDT_S_T2 | 99.63 |
| PDT_G_T2 | 99.47 |
| PDT_F_T2 | 100.00 |
| PDT_S_T1 | - |
| PDT_G_T1 | 99.56 |
| PDT_F_T1 | 99.57 |

**Supplementary Figure 3A:** Flowchart explaining the combinations of cutoffs for the gene NP_644816.1. Tick and cross represent subsequent flow of program as per the phyletic distribution threshold (PDT) values for different taxonomic levels. Final decision is marked with red dotted box. On the right side, table shows different PDT values at species, genus and family levels.

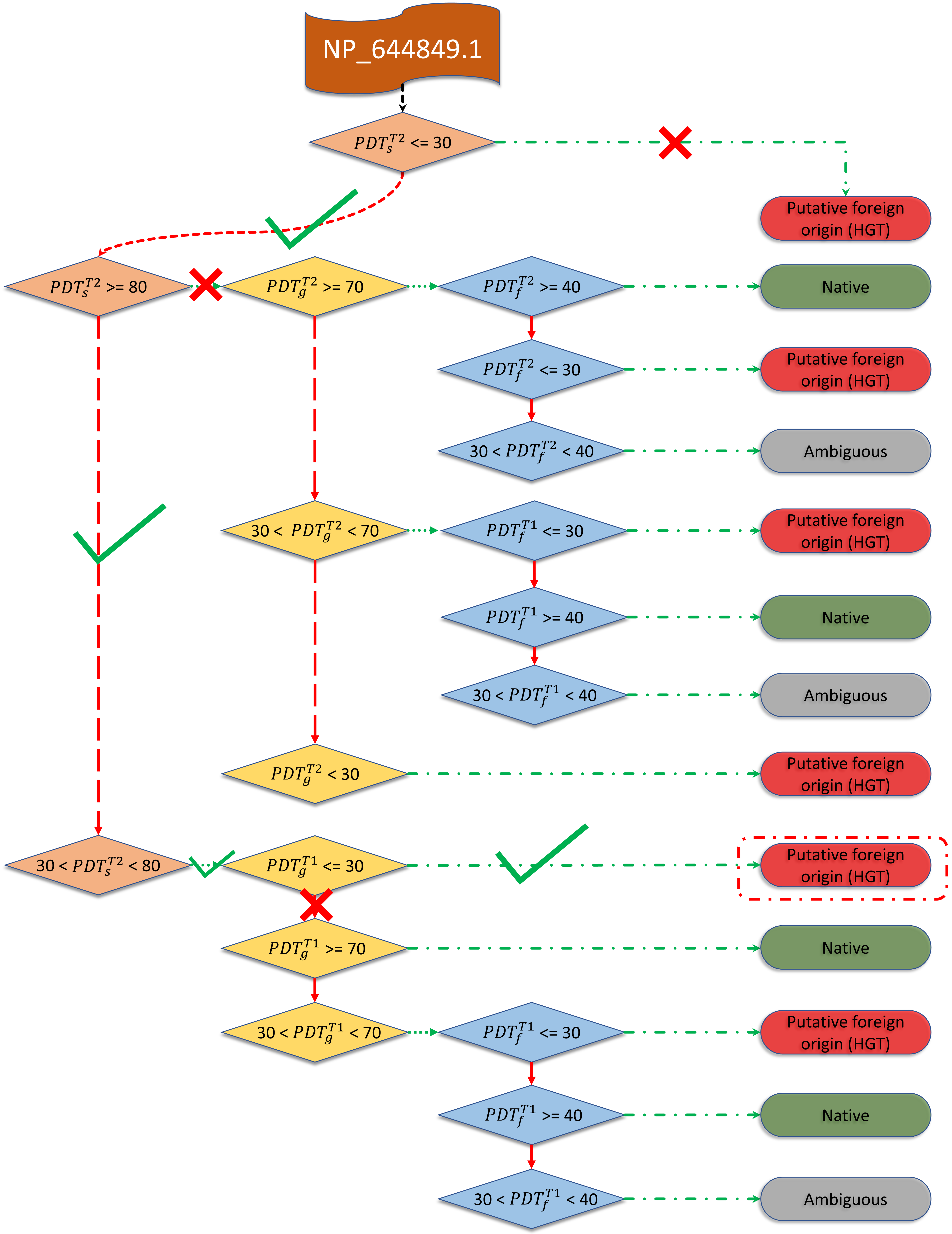


| Phyletic Distribution Threshold (PDT) | NP_644849.1 |
|---|---|
| PDT_S_T2 | 32.15 |
| PDT_G_T2 | 24.20 |
| PDT_F_T2 | 7.14 |
| PDT_S_T1 | - |
| PDT_G_T1 | 28.87 |
| PDT_F_T1 | 32.32 |

**Supplementary Figure 3B:** Flowchart explaining the combinations of cutoffs for the gene NP_644849.1. Tick and cross represent subsequent flow of program as per the phyletic distribution threshold (PDT) values for different taxonomic levels. Final decision is marked with red dotted box. On the right side, table shows different PDT values at species, genus and family levels.
